# A mathematical model captures the role of adenyl cyclase Cyr1 and guanidine exchange factor Ira2 in creating a growth‐to‐hyphal bistable switch in *Candida albicans*

**DOI:** 10.1002/2211-5463.13470

**Published:** 2022-08-30

**Authors:** K Sriram

**Affiliations:** ^1^ Department of Computational Biology, Center for Computational Biology IIIT‐Delhi India

**Keywords:** bistable, *Candida albicans*, ODE, positive feedback, Ras signaling pathway, yeast‐hyphal switching

## Abstract

Recent biochemical experiments have indicated that in *Candida albicans*, a commensal fungal pathogen, the Ras signaling pathway plays a significant role in the yeast‐to‐hyphal transition; specifically, two enzymes in this pathway, Adenyl Cyclase Cyr1 and GTPase activating protein Ira2, facilitate this transition, in the presence of energy sensor ATP. However, the precise mechanism by which protein interactions between Ira2 and Cyr1 and the energy sensor ATP result in the yeast‐to‐hyphal transition and create a switch‐like process are unknown. We propose a new set of biochemical reaction steps that captures all the essential interactions between Ira2, Cyr1, and ATP in the Ras pathway. With the help of chemical reaction network theory, we demonstrate that this set of biochemical reaction steps results in bistability. Further, bifurcation analysis of the differential equations based on this set of reaction steps supports the existence of a bistable switch, and this switch may act as a checkpoint mechanism for the promotion of growth‐to‐hyphal transition in *C. albicans*.

## Graphical abstract

The cause for yeast‐hyphal transition in recent experiments on *C. albicans* implied the existence of interactions between the energy sensor ATP, adenyl cyclase CYR1, and the GTPase activating factor IRA2 in the Ras signaling pathway. Considering these interactions, we built a Ras network and the corresponding ODE model to show the existence of a bistable switch, which we attribute to the morphological transition.

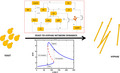

**Abbreviations**
AC: adenyl cyclase
*C. albicans*
: *Candida albicans*
CRNT: chemical reaction network theory
GAP: GTPase activating protein
GEF: guanine nucleotide exchange factor
MM: Michaelis–Menten
ODE: ordinary differential equations
PDE: phosphodiesterases
RD: Ras‐GDP
RT: Ras‐GTP
xppaut
: X windows‐Phase‐Plane‐AUTomatic bifurcation software

*Candida albicans* is an opportunistic, commensal fungal pathogen that can cause life‐threatening disease in people with compromised immune systems. They can thrive in many hosts by adapting themselves to any conditions for their survival. *C. albicans* can exist in three morphologies: yeast‐like, hyphae, and pseudo‐hyphae, with the last two together commonly referred to as filamentous morphology. Interestingly, *C. albicans* turns out to be virulent only when switching from yeast‐to‐hyphae is favorable under a given host condition. If a strain is either locked into a yeast or filamentous form, it fails to mount any infection, and both *in vitro* and *in vivo* infection models support it. It is the morphological switching rather than specific morphology *per se* that is important for virulence [1, 2, 3]. Therefore, studying the cause of switching from the molecular mechanistic perspective is beneficial in identifying efficacious antifungal drug targets.

Recent biochemical experiments have implicated the Ras signaling pathway as one of the major players in effecting morphological switching [4, 5]. In the Ras pathway, the two small G proteins, Ras1 and Ras2, undergo a conformational switch in the presence of GTP and GDP. In response to environmental stimuli, two opposing enzymes tightly control the Ras activity: (a) Cdc25 is the guanine nucleotide exchange factor (GEF) that activates G protein by exchanging GDP with GTP, and (b) Ira2 is the GTPase‐activator protein (GAP) that inactivates Ras by hydrolyzing GTP to GDP. Activated Ras‐GTP interacts with adenyl cyclase (AC) Cyr1 to stimulate cAMP production from ATP. ATP is generated from the metabolic reactions and transported from mitochondria to the site of action. cAMP then derepresses PKA; the protein kinase A. PKA is a tetramer consisting of two catalytic and two regulatory subunits. Together, these subunits are inactive, but cAMP binding to the regulatory subunit frees the catalytic subunit for further downstream cellular processes. Low‐ and high‐affinity phosphodiesterases, namely PDE1 and PDE2, respectively, downregulates cAMP production. They form a negative feedback loop with cAMP to limit the formation of hyphal phenotypes [6, 7].

Recently, Silao et al. [3] and Grahl et al. [8] biochemical experiments have provided evidence that ATP released from mitochondria, AC Cyr1, and guanidine exchange factor Ira2 are the critical regulators of the Ras pathway that controls the morphological switching in *C. albicans*. They show in their experiments that depletion of ATP by its inhibitors results in less Ras‐GTP activity and, as a result, arrests yeast‐to‐hyphal formation. Further, they show that Ras activity requires Ira2 but not Cdc25. Based on these observations, they proposed that (a) During ATP depletion, Cyr1, although a downstream regulator, together with upstream regulator Ira2, downregulates Ras‐GTP, and (b) Ira2‐Cyr1‐dependent repression with intracellular ATP concentration acts as a checkpoint for the yeast to decide to switch between the reverse virulent hyphal and nonvirulent yeast forms [3]. During the transition to hyphal form, with sufficient ATP present to drive the process, Ras‐GTP and cAMP levels were also present at a higher level. Although this explanation is attractive and based on experimental evidence, they have not proposed any biochemical reactions, and it is not clear how regulation between Ira2 and Cyr1 in the Ras network brings about morphological switching. Further, Rocha et al. [7] showed that deletion of Cyr1 itself is sufficient to arrest the yeast‐to‐hyphal transition and the virulence. Also, the transcription factors (TFs) and other pathways like MAPK depend on the Ras pathway for morphological transition and virulence. For example, Lebrer et al. [6] showed that among Ras and MAPK pathways, the intervening Ras pathway leads directly to changes in the morphologies of *C. albicans*. TFs also play a critical role in facilitating the yeast‐to‐hyphal transition. For example, *ras1*, *cyr1*, or *pka1* mutation leads to a loss of function in biofilm formation, and overexpression of EFG1, an important TF, leads to the rescue of hyphal elongation in biofilm formation [5]. Similarly, regulation of TFs Tec1 and Bcr1 by the Ras pathway provides an understanding of the morphological changes brought about in *C. albicans*. Environmental cues also trigger selective signaling pathways that mediate yeast‐to‐hyphal transitions and *vice versa*. For example, nutrient limitation triggers the MAPK pathway, Alkaline pH induces the Rim101 pathway, and elevated temperature triggers HSP90/cAMP‐PKA pathway. All the above pathways trigger yeast‐to‐hyphal morphogenesis. Hyperosmotic stress conditions trigger the HOG pathway, while quorum sensing molecules trigger cAMP‐PKA pathways. These two pathways repress hyphal growth [2, 5].

In this work we provide a new set of biochemical reaction steps to explain the occurrence of morphological switching. Based on these, a new set of reaction steps, we capture the dynamics of the Ras/Cyr1/PKA/cAMP pathway through ordinary differential equation models. The reaction steps are broadly based on the experiments of Grah et al. [8] that shows the importance of Ira2‐Cyr1‐ATP interactions in bringing about yeast‐to‐hyphal switching. ATP, an energy sensor, plays a vital role, and by its very presence or absence, the strength of Ira2‐Cyr1 interactions in *C. albicans* varies, and under favorable conditions the yeast‐to‐hyphal transition takes place.

We build our model of the Ras signaling pathway by only considering the mass action kinetic rate laws rather than the Michaelis–Menten (MM) type of kinetics. Since Ira2‐Cyr1 are enzyme–enzyme interactions, modeling by MM laws may be inappropriate as, it may not satisfy the requirement of substrate concentration ≫ enzymatic concentration [9, 10].

We also apply chemical reaction network theory (CRNT) to identify whether our network can give rise to bistability without having to rely on kinetic parameters [11]. We are specifically interested in bistable dynamics because, based on experiments, we posit that the Ras pathway may create switch‐like dynamics that reflect the yeast‐to‐hyphal transition. Bistability is seen in many signaling [12, 13, 14], metabolic [15], and gene regulatory motifs [16] that have specific structural requirement like positive feedback, substrate inhibition, etc. However, for the first time, Markevich et al. [17] have shown the presence of bistability in the MAPK signaling cascade pathway even though there is no apparent positive feedback loop in the motif. Recently, Li et al. [18] proposed a new bistable mechanism for a network with no apparent positive feedback loop. Their study on the mRNA–miRNA interaction showed that noncanonical feedback is responsible for bistability. The network structure has stoichiometric inhibition and degradation reactions as the main components. When these components regulate asymmetrically, bistability occurs. Bistable or a toggle switch is a response when the continuous input signal provides a discontinuous output ON/OFF response. The input signal strength moves the system either to OFF or ON states, and the system's history decides which state the system can finally rest in [19].

The following section explains all the biochemical steps based on the network structure of the Ras/cAMP/PKA network (Fig. 1). We also employ CRNT to identify the existence of bistability, and, if it exists, we support it through bifurcation analysis for further analysis.

**Fig. 1:**
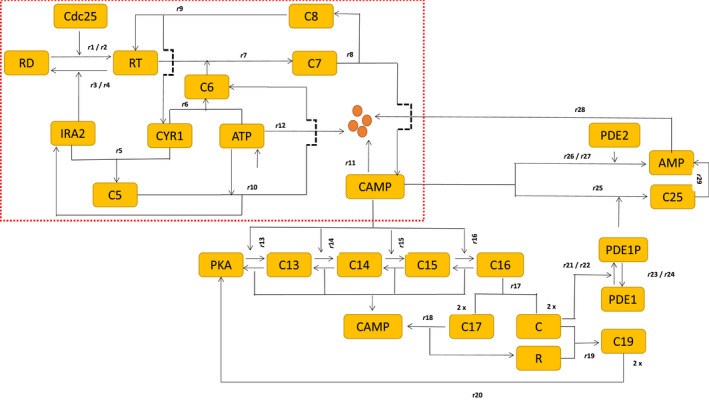
Biological network of Ras‐GTP pathway. Yellow boxes with names are protein molecules. We first start on the top left of the circuit inside the dotted box with RD, a short form for Ras‐GDP, and RT for Ras‐GTP. The interactions inside the dotted box show the new reaction for the interaction between Ira2 and Cyr1, ATP and Cyr1, which plays a vital role in forming cAMP. Subsequently, the other module outside the box is the derepression of PKA by cAMP that releases the catalytic C part of the PKA and downregulates cAMP through phosphodiesteRases PDE‐1 and 2 to form a negative feedback loop. The *ri*'s represent the rates of the reactions based on mass action kinetic laws. In detail, Ras‐GDP is converted to RT, the Ras‐GTP, by the enzyme cdc25. The enzyme Ira2 carries out the reverse reaction. Cyr1, the enzyme adenyl‐cyclase, binds to Ira2 to form a complex C5 to arrest the conversion of Ras‐GTP to Ras‐GDP, and thereby reduce its activity. Cyr1 also binds to ATP, which forms an inactive complex C6. This inactive complex C6 binds to RT to form a trimer C7 that facilitates cAMP formation. The competition between complexes C5 and C6 decides the RT activity and cAMP formation. This tug‐of‐war for RTP by C5 and C6 leads to a positive feedback loop that generates bistability for the choice of parameters. Both cAMP and ATP undergo first‐order exponential degradation. *ri* are the rates of the reaction. The only role of cAMP is to derepress the catalytic part of PKA from the regulatory part by a sequence of steps that forms complexes C13–C16. The final complex C16 dissociates PKA and ejects out cAMP. The catalytic part of PKA involves cAMP degradation through negative feedback that involves low‐affinity phosphodiesteRases‐1 (PDE1) and gets converted to AMP. The high‐affinity phosphodiester Rase‐2 (PDE2) also converts cAMP to AMP to maintain the cAMP homeostasis. Note that we do not provide the details of the enzymatic conversion of PDE1 to PDE1P and have given only the rates *ri*'s.

## Methods

### Ras network, their corresponding biochemical steps, and ODE

The Ras network broadly consists of two modules. The dotted box in Fig. 1 is module‐1 and the rest are module‐2. Module‐1 is a set of reactions describing cAMP production starting from Ras‐GDP. Module‐2 is a set of reactions describing the cAMP conversion to AMP through PKA and PDEs. The reaction steps R1–R12 below provide the details of module‐1 and the reaction steps R13–R29 are module‐2.

We base all the reactions below on the laws of mass action kinetics. We map the biochemical steps directly from the network given in Fig. 1. kf*i*'s and kr*i*'s are *i*th forward and backward reactions, respectively. R*i*'s are the reactions of the *i*th biochemical step, and *ri*'s are the corresponding rates of the reaction.

R1–R4: The first step involves the conformational switch of Ras protein from an inactive Ras‐GDP (RD) to active Ras‐GTP (RTP) in response to environmental stimuli brought about by the guanyl‐nucleotide exchange factor (GEF) Cdc25. Ira2, the GTPase activating protein (GAP), carries out the reverse reaction [20]. In reactions R1–R4, we show the enzymatic reactions in the stepwise MM form. We provide the corresponding rates, *r*1–*r*4, on the right side. Note again that we use short form RD for Ras‐GDP and RTP for Ras‐GTP. We maintain this throughout the article.

$$R1:\;\mathrm{RD}+\mathrm{CD}25\overset{\mathrm{kf}1}{\underset{\mathrm{kr}1}{⇌}}C1\;r1=\mathrm{kf}1\left[\mathrm{CD}25\right]\left[\mathrm{RD}\right]-\mathrm{kr}1\left[C1\right],$$

$$R2:\;C1\overset{\mathrm{kf}2}{\to }\mathrm{RTP}+\mathrm{CD}25\;r2=\mathrm{kf}2\left[C1\right],$$

$$R3:\;\mathrm{RTP}+\mathrm{Ira}2\overset{\mathrm{kf}3}{\underset{\mathrm{kr}3}{⇌}}C3\;r3=\mathrm{kf}3\left[\mathrm{RTP}\right]\left[\mathrm{Ira}2\right]-\mathrm{kr}3\left[C3\right],$$

$$R4:\;C3\overset{\mathrm{kf}4}{\to }\mathrm{RD}+\mathrm{Ira}2\;r4=\mathrm{kf}4\left[C3\right].$$

R5–R7: The enzyme AC, Cyr1, is a vital hub in the Ras pathway [21] that promotes virulence in *C. albicans* [7]. Although Cyr1 is a downstream effector in the conversion of ATP to cAMP, recent experiments indicate that in the absence of ATP, Cyr1 may act upstream in the pathway and regulates Ras activity in *C. albicans* ([3], see fig. 9 of [8]). As a result, Ira2 and ATP, if present in sufficient quantity, compete to bind to Cyr1 and form complexes C5 and C6, respectively. This antagonistic interaction sets up the RTP regulation; i.e. on the one hand, a sufficient amount of ATP first captures Cyr1 and then sequesters RTP to convert ATP to cAMP, while on the other hand, Ira2 retards RTP progression by converting RTP to inactive RD. The fate of the RTP level now depends on the strength of the two complexes, C5 and C6. When RTP binds to complex C6, it leads to the cAMP formation through a complex C7. We capture these reactions below.

$$R5:\;\mathrm{Ira}2+\mathrm{Cyr}1\overset{\mathrm{kf}5}{\underset{\mathrm{kr}5}{⇌}}C5\;r5=\mathrm{kf}5\left[\mathrm{Ira}2\right]\left[\mathrm{Cyr}1\right]-\mathrm{kr}5\left[C5\right],$$

$$R6:\;\mathrm{ATP}+\mathrm{Cyr}1\overset{\mathrm{kf}6}{\underset{\mathrm{kr}6}{⇌}}C6\;r6=\mathrm{kf}6\left[\mathrm{ATP}\right]\left[\mathrm{Cyr}1\right]-\mathrm{kr}6\left[C6\right],$$

$$R7:\;C6+\mathrm{RTP}\overset{\mathrm{kf}7}{\underset{\mathrm{kr}7}{⇋}}C7\;r7=\mathrm{kf}7\left[C6\right]\left[\mathrm{RTP}\right]-\mathrm{kr}7\left[C7\right].$$

R8–R10: The next step involves the conversion of complex C7 from reaction R7 to CAMP and another complex, C8. The complex C8 is composed of RTP, and Cyr1 gets dissociated in the subsequent step. The Cyr1 in the free form is inactive, and therefore to be active it has to complex with the RTP to bind to ATP to convert to cAMP. There is evidence that Cyr1 behaves like a scaffold to assemble ATP and Ras‐GTP [22]. We assume that the complex C5, in reaction R5, recruits ATP to form a scaffold transiently to release only Ira2 and form a complex C6. This reaction exchanges Ira2 and ATP between the complexes C5 and C6. Depending on the availability of Ira2 or ATP, either reaction R7 or R5 takes precedence. For example, in the presence of abundant ATP, reactions R6 and R7 proceed to generate cAMP and lead to the hyphal formation.

$$R8:\;C7\overset{\mathrm{kf}8}{\underset{\mathrm{kr}8}{⇌}}\text{cAMP}+C8\;r8=\mathrm{kf}8\left[C7\right]-\mathrm{kr}8\left[C8\right]\left[\text{CAMP}\right],$$

$$R9:\;C8\overset{\mathrm{kf}9}{⇌}\mathrm{RTP}+\mathrm{Cyr}1\;r9=\mathrm{kf}9\left[C8\right]-\mathrm{kr}9\left[\mathrm{RTP}\right]\left[\mathrm{Cyr}1\right],$$

$$R10:\;C5+\mathrm{ATP}\overset{\mathrm{kf}10}{\to }C6+\mathrm{Ira}2\;r10=\mathrm{kf}10\left[C5\right]\left[\mathrm{ATP}\right]-\mathrm{kr}10\left[C6\right]\left[\mathrm{Ira}2\right].$$

R11–R12: We assume that cAMP undergoes a first‐order exponential degradation besides being negatively regulated by phosphodiesteRases PDE1 and 2. ATP produced from the metabolic reactions is also assumed to undergo exponential degradation. The reaction steps are:

$$R11:\;\text{CAMP}\overset{\mathrm{kf}10}{\to }0\;r11=\mathrm{kf}11\left[\text{CAMP}\right],$$

$$R12:\;0\overset{\mathrm{kf}8}{\underset{\mathrm{kr}8}{⇌}}\mathrm{ATP}\;r12=\mathrm{kf}12-\mathrm{kr}12\left[\mathrm{ATP}\right].$$

In both R11 and R12 reactions, ‘0’ indicates production or loss. If ‘0’ is present to the left of the reaction arrow, it indicates production, whereas on the right of the reaction arrow it indicates a loss. In CRNT terminology, this denotes the inflow and outflow of the reaction. Also, it indicates an open system.

Reactions R5–R12 are the new set of reaction steps we will use to explain the occurrence of the yeast‐to‐hyphal switch. The important reactions R5–R12 are new because not only are they not from the preexisting model, but they are also very specific to the problem at hand. The basis of these reactions is from the Grahl et al. article [8]. In their article, they provided in fig. 9 the cartoon of IRA2, ATP, and RAS‐GTP regulations without giving any details of the full reactions. We converted this cartoon into a series of step‐wise reversible reactions based on the laws of mass action kinetics. These reactions are specific to *C. albicans*, which forms the backbone of this work. The rest of the reactions below explain the negative feedback loop between PKA and cAMP that releases the catalytic from regulatory subunits. All the steps that follow are from the earlier work [23].

R13–R16: Presently, we only know that cAMP activates PKA. PKA is a tetramer of two catalytic and two regulatory subunits. cAMP bind to all four subunits of PKA, and we provide the stepwise addition of cAMP to PKA. The complexes C13–C15 capture the various stoichiometries of cAMP binding to PKA.

$$R13:\;\text{CAMP}+\mathrm{PKA}\overset{\mathrm{kf}13}{\underset{\mathrm{kr}13}{⇌}}C13\;r13=\mathrm{kf}13\left[\text{CAMP}\right]\left[\mathrm{PKA}\right]-\mathrm{kr}13\left[C13\right],$$

$$R14:\;C13+\text{CAMP}\overset{\mathrm{kf}14}{\underset{\mathrm{kr}14}{⇌}}C14\;r14=\mathrm{kf}14\left[C13\right]\left[\text{CAMP}\right]-\mathrm{kr}14\left[C14\right],$$

$$R15:\;C14+\text{CAMP}\overset{\mathrm{kf}15}{\underset{\mathrm{kr}15}{⇌}}C15\;r15=\mathrm{kf}15\left[C14\right]\left[\text{CAMP}\right]-\mathrm{kr}15\left[C15\right],$$

$$R16:\;C15+\text{CAMP}\overset{\mathrm{kf}16}{\underset{\mathrm{kr}16}{⇌}}C16\;r16=\mathrm{kf}16\left[C15\right]\left[\text{CAMP}\right]-\mathrm{kr}16\left[C16\right].$$

R17–R20: The steps below capture the outcome of the complex C16 that derepresses PKA and release two catalytic subunits from the two regulatory subunits. These subunits can also come together to form full PKA.

$$R17:\;C16\overset{\mathrm{kf}17}{\underset{\mathrm{kr}17}{⇌}}2C+2C17\;r17=\mathrm{kf}17\left[C16\right]-\mathrm{kr}17{\left[C\right]}^{2}{\left[C17\right]}^{2},$$

$$R18:\;C17\overset{\mathrm{kf}18}{\to }R+\text{CAMP}\;r18=\mathrm{kf}18\left[C17\right],$$

$$R19:\;R+C\overset{\mathrm{kf}20}{\underset{\mathrm{kr}19}{⇌}}C19\;r19=\mathrm{kf}19\left[R\right]\left[C\right]-\mathrm{kr}19\left[C19\right],$$

$$R20:\;2C19\overset{\mathrm{kf}20}{\underset{\mathrm{kr}20}{⇌}}\mathrm{PKA}\;r20=\mathrm{kf}20{\left[C19\right]}^{2}-\mathrm{kr}20\left[\mathrm{PKA}\right].$$

R21–R29: The final reactions below capture the catalytic degradation of cAMP by negative feedback regulation of phosphodiesteRase‐1 (PDE1) by PKA. This degradation is independent of the enzymatic degradation carried out by high‐affinity PDE‐2 to convert cAMP to AMP. The final product AMP also undergoes first‐order degradation.

$$R21:\;C+\mathrm{PDE}1\overset{\mathrm{kf}21}{\underset{\mathrm{kr}21}{⇌}}C21\;r21=\mathrm{kf}21\left[C\right]\left[\mathrm{PDE}1\right]-\mathrm{kr}21\left[C21\right],$$

$$R22:\;C21\overset{\mathrm{kf}22}{\to }\mathrm{PDE}1P+C\;r22=\mathrm{kf}22\left[C21\right],$$

$$R23:\;\mathrm{PDE}1P+\mathrm{PPA}\overset{\mathrm{kf}23}{\underset{\mathrm{kr}23}{⇌}}C23\;r23=\mathrm{kf}23\left[\mathrm{PDE}1P\right]\left[\mathrm{PPA}\right]-\mathrm{kr}23\left[C23\right],$$

$$R24:\;C23\overset{\mathrm{kf}24}{\to }\mathrm{PDE}1+\mathrm{PPA}\;r24=\mathrm{kf}24\left[C23\right],$$

$$R25:\;\mathrm{PDE}1P+\text{CAMP}\overset{\mathrm{kf}25}{\to }C25\;r25=\mathrm{kf}25\left[\text{CAMP}\right]\left[\mathrm{PDE}1P\right]-\mathrm{kr}25\left[C25\right],$$

$$R26:\;\text{CAMP}+\mathrm{PDE}2\overset{\mathrm{kf}26}{\underset{\mathrm{kr}26}{⇌}}C26\;r26=\mathrm{kf}26\left[\text{CAMP}\right]\left[\mathrm{PDE}2\right]-\mathrm{kr}26\left[C26\right],$$

$$R27:\;C26\overset{\mathrm{kf}27}{\to }\mathrm{AMP}+\mathrm{PDE}2\;r27=\mathrm{kf}27\left[C26\right],$$

$$R28:\;\mathrm{AMP}\overset{\mathrm{kf}28}{\to }0\;r28=\mathrm{kf}28\left[\mathrm{AMP}\right],$$

$$R29:\;C25\overset{\mathrm{kf}29}{\to }\mathrm{AMP}+\mathrm{PDE}1P\;r29=\mathrm{kf}29\left[C25\right].$$

The corresponding differential equations are:

(1) $$\frac{d\left[\mathrm{RTP}\right]}{dt}=r2-r3-r7+r9,$$

(2) $$\frac{d\left[\mathrm{ATP}\right]}{dt}=-r6-r10+r12,$$

(3) dCAMPdt=r8+r18−r11+r13+r14+r15+r16+r25+r26,

(4) $$\frac{d\left[\mathrm{PDE}1P\right]}{dt}=r22-r23-r25+r29,$$

(5) $$\frac{d\left[\mathrm{AMP}\right]}{dt}=r27-r28+r29,$$

(6) $$\frac{d\left[C1\right]}{dt}=r1-r2,$$

(7) $$\frac{d\left[C3\right]}{dt}=r3-r4,$$

(8) $$\frac{d\left[C5\right]}{dt}=r5-r10,$$

(9) $$\frac{d\left[C6\right]}{dt}=r6-r7+r10,$$

(10) $$\frac{d\left[C8\right]}{dt}=r8-r9,$$

(11) $$\frac{d\left[C13\right]}{dt}=r13-r14,$$

(12) $$\frac{d\left[C14\right]}{dt}=r14-r15,$$

(13) $$\frac{d\left[C15\right]}{dt}=r15-r16,$$

(14) $$\frac{d\left[C16\right]}{dt}=r16-r17,$$

(15) $$\frac{d\left[C17\right]}{dt}=2*r17-r18,$$

(16) $$\frac{d\left[C19\right]}{dt}=r19-2*r20,$$

(17) $$\frac{d\left[C21\right]}{dt}=r21-r22,$$

(18) $$\frac{d\left[C23\right]}{dt}=r23-r24,$$

(19) $$\frac{d\left[C25\right]}{dt}=r25-r29,$$

(20) $$\frac{\text{d}\left[\text{C}26\right]}{\text{d}t}=r26-r27.$$

To determine the conservation relationship, we apply Gaussian elimination to the reaction rate stoichiometric matrix obtained from ODEs. Gaussian elimination gives linearly independent and dependent variables [24] from which we obtained the conservation relationship. We provide the MatLab code (MathWorks, Natick, MA, USA) (Appendix S1) and the stoichiometric matrix file in Appendix S2. The 10 conserved relationships are:

$$\begin{array}{c} \left[\mathrm{CD}25\right]=\mathrm{CD}25T-\left[C1\right],\\ \left[\mathrm{Ira}2\right]=\mathrm{Ira}2T-\left(\left[C3\right]+\left[C5\right]\right),\\ \left[\mathrm{Cyr}1\right]=\mathrm{Cyr}1T-\left(\left[C5\right]+\left[C6\right]+\left[C7\right]\right),\\ \left[\mathrm{RD}\right]=\text{RasT}-\left(\left[C1\right]+\left[C3\right]+\left[C7\right]+\left[C8\right]+\left[\mathrm{RTP}\right]\right),\\ \left[\mathrm{PDE}1\right]=\mathrm{PDE}1T-\left(\left[\mathrm{PDE}1P\right]+\left[C21\right]+\left[C23\right]+\left[C25\right]\right),\\ \left[\mathrm{PDE}2\right]=\mathrm{PDE}2T-\left[C26\right],\\ \left[C\right]=\mathrm{CT}-\left(2*\left[C13\right]+2*\left[C14\right]+2*\left[C15\right]+2*\left[C16\right]+\left[C19\right]+C21+2*\left[\mathrm{PKA}\right]\right),\\ \left[R\right]=\mathrm{RT}-\left(2*\left[C13\right]+2*\left[C14\right]+2*\left[C15\right]+2*\left[C16\right]+\left[C19\right]+\left[C17\right]+2*\left[\mathrm{PKA}\right]\right),\\ \left[\mathrm{PKA}\right]=\frac{1}{4}*(\text{PKAT}-(4*\left[C13\right]+4*\left[C14\right]+4*\left[C15\right]+4*\left[C16\right]+2*\left[C19\right]\\ \;+\left[C17\right]+\left[C21\right]+\left[R\right]+\left[C\right])),\\ \left[\mathrm{PPA}\right]=\text{PPAT}-\left[C23\right]. \end{array}$$

There are overall 20 reactions and 10 conservation relationships. Since R and C are regulatory and catalytic subunits of PKA, the total PKA ([PKAT]) = CT + RT is a constant.

### Structural analysis using CRNT

Since the kinetic parameters of the model are not known from the *C. albicans* experiments to analyze any further, we apply CRNT to determine the structural properties of the network. CRNT provides a connection between the network structure and dynamics, independent of the kinetic parameters. The specific dynamic we look for in this network is bistability. Bistability occurs when two stable equilibrium states, namely nodes, are separated by an unstable equilibrium state saddle. When saddle and node collide, it gives rise to saddle‐node bifurcation. Bistability can be irreversible or reversible (toggle switch). We are specifically interested in the toggle switch dynamics because the proposed cooperative interactions of Cyr1 and Ira2 may provide positive feedback that acts as a checkpoint in yeast‐to‐filamentous growth [3]. Craciun et al. [11] provide the necessary and sufficient conditions for a network to exhibit multistability. Further, the toolbox to analyze the network is freely available (https://cbe.osu.edu/chemical‐reaction‐network‐theory#toolbox), and this tool takes as input the elementary chemical reactions based on mass action kinetics and provides output information related to the existence of multistability. When we input the reactions R1–R29, the CRNT toolbox indicates that the network structure can exhibit multistability. Besides, we also obtain other information, like deficiency of the network based on the number of complexes (*n*), linkage class (*l*), and rank of the stoichiometric matrix (*r*) from the toolbox. The deficiency δ = *n* − *l* − *r* is > 1 for our network structure; i.e. 7.

Using the CRNT toolbox, we also looked for redundant reactions in the minimal model that do not contribute to multistability. The minimal model based on the laws of mass action kinetics consists of reactions R1–R12. We eliminated each reaction one by one, checked for the existence of multistability, and found all the reactions to be important. Next, to the minimal model we added the reactions R13–R28, namely, CAMP regulation by PKA, as shown below in the dotted box of Fig. 1, and carried out a similar exercise. Among the 29 reactions, we find that all the reactions are required for the network structure to exhibit multistability except for cAMP degradation (reaction R11). The cAMP appears redundant due to the inclusion of AMP loss, which we obtained from CAMP conversion. Therefore, all the reactions in the network are important for bistability. In summary, we find that almost all the reactions are important for the network to exhibit multistability. Since ATP, the energy sensor, plays a strong role in the yeast‐to‐hyphal transition, kf12, the production constant, is taken as a bifurcation parameter to carry out various analyses. Besides, we also choose CYR1T, the network's hub, and IRA2T, which competes with CYR1T for RAS‐GTP, to be the other bifurcation parameters to understand the morphological transition dynamics of *C. albicans*.

To perform bifurcation analysis, We used most of the kinetic constants (all values > 0) obtained from the toolbox (Table 1), and, wherever possible, we back‐calculate to get the rest of the parameters as given in Table 2. We took μm as the unit for chemical species and seconds for time. We provide the .NET file to run our network using the CRNT toolbox in Appendix S3. We have not further fine‐tuned the parameters obtained from the CRNT toolbox and use them as provided by the toolbox. In the next section we discuss and interpret the results obtained from the bifurcation analysis using the freely available software xppaut [25].

**Table 1:** Kinetic constants of the full Ras pathway model from the CRNT toolbox.

kf1 = 2.6702E‐2 μm ^−1^·s^−1^	kr1 = 0.22140275 s^−1^	kf2 = 0.22140275 s^−1^
kf3 = 0.20554648 μm ^−1^·s^−1^	kr3 = 0.22140275 s^−1^	kf4 = 0.22140275 s^−1^
kf5 = 0.18971829 μm ^−1^·s^−1^	kr5 = 2.6163E‐2 s^−1^	kf6 = 3.7537582 μm ^−1^·s^−1^
kr6 = 7.4770E‐2 s^−1^	kf7 = 3.6846E‐2 μm ^−1^·s^−1^	kr7 = 0.1039351 s^−1^
kf8 = 0.79413092 s^−1^	kf9 = 12.486667 s^−1^	kr9 = 2.7294819 μm ^−1^·s^−1^
kf10 = 1.4802E‐2 μm ^−1^·s^−1^	kr10 = 4.0322E‐3 μm ^−1^·s^−1^	kf11 = 1 s^−1^
kf12 = 9.5940921 μm s^−1^	kr12 = 1 s^−1^	kf13 = 190.24416 μm ^−1^·s^−1^
kr13 = 78.310111 s^−1^	kf14 = 77.679886 μm ^−1^·s^−1^	kr14 = 11.430544 s^−1^
kf15 = 9.6497898 μm ^−1^·s^−1^	kr15 = 6.2403657 s^−1^	kf16 = 2.2702379 μm ^−1^·s^−1^
kr16 = 1.7830E‐2 s^−1^	kf17 = 5.3671E‐2 s^−1^	kr17 = 2.0544E‐2 μm ^−3^·s^−1^
kf18 = 0.75147295 s^−1^	kf19 = 4.7825E‐2 μm ^−1^·s^−1^	kr19 = 2.6692966 μm ^−1^·s^−1^
kf20 = 103.14004 μm ^−1^·s^−1^	kr20 = 180.79459 s^−1^	kf21 = 5.0883E‐2 μm ^−1^·s^−1^
kr21 = 0.49182469 μm ^−1^ s^−1^	kf22 = 0.49182469 s^−1^	kf23 = 0.12346798 μm ^−1^·s^−1^
kr23 = 0.49182469 s^−1^	kf24 = 0.49182469 s^−1^	kf25 = 1.9228563 μm ^−1^·s^−1^
kr25 = 5.8292E‐2 s^−1^	kf26 = 2.815359 μm ^−1^·s^−1^	kr26 = 9.5162E‐2 s^−1^
kf27 = 0.16911965 s^−1^	kf28 = 1 s^−1^	kf29 = 0.11449047 s^−1^

**Table 2:** Mix of estimated and CRNT kinetic constants for the full Ras pathway model.

kf1 = 2.6702E‐2 μm ^−1^·s^−1^	kr1 = 0.0085 s^−1^	kf2 = 0.0050 s^−1^
kf3 = 0.20554648 μm ^−1^·s^−1^	kr3 = 0.0077 s^−1^	kf4 = 0.2000 s^−1^
kf5 = 0.18971829 μm ^−1^·s^−1^	kr5 = 2.6163E‐2 s^−1^	kf6 = 3.7537582 μm ^−1^·s^−1^
kr6 = 7.4770E‐2 s^−1^	kf7 = 3.6846E‐2 μm ^−1^·s^−1^	kr7 = 0.1039351 s^−1^
kf8 = 0.79413092 s^−1^	kf9 = 12.486667 s^−1^	kr9 = 2.7294819 μm ^−1^·s^−1^
kf10 = 1.4802E‐2 μm ^−1^·s^−1^	kr10 = 4.0322E‐3 μm ^−1^·s^−1^	kf11 = 1 s^−1^
kf12 = 9.5940921 μm·s^−1^	kr12 = 1 s^−1^	kf13 = 0.00009 μm ^−1^·s^−1^
kr13 = 33 s^−1^	kf14 = 0.00009 μm ^−1^·s^−1^	kr14 = 33 s^−1^
kf15 = 0.000125 μm ^−1^·s^−1^	kr15 = 110 s^−1^	kf16 = 0.000125 μm ^−1^·s^−1^
kr16 = 32.5 s^−1^	kf17 = 60 s^−1^	kr17 = 0.00003 μm ^−3^·s^−1^
kf18 = 0.75147295 s^−1^	kf19 = 4.7825E‐2 μm ^−1^·s^−1^	kr19 = 2.6692966 μm ^−1^·s^−1^
kf20 = 103.14004 μm ^−1^·s^−1^	kr20 = 180.79459 s^−1^	kf21 = 5.0883E‐2 μm ^−1^·s^−1^
kr21 = 0.2016 μm ^−1^·s^−1^	kf22 = 0.18 s^−1^	kf23 = 0.12346798 μm ^−1^·s^−1^
kr23 = 0.49182469 s^−1^	kf24 = 0.49182469	kf25 = 1.9228563 μm ^−1^·s^−1^
kr25 = 34.1514 s^−1^	kf26 = 4 μm ^−1^·s^−1^	kr26 = 34.1514 s^−1^
kf27 = 8 s^−1^	kf28 = 1 s^−1^	kf29 = 4 s^−1^

### Parameter estimation

We combed the literature to extract parameters for the Ras pathway. However, kinetic parameters from *C. albicans* experiments are not readily available. Time course data are also not available. We found that most of the Ras pathway models in the literature are in MM form or the combination of MM and MAK forms [26, 27, 28]. Thus, most of the reactions have enzyme kinetics parameters, namely *V*_max_ and KM, but hardly any association or dissociation constants suitable for mass action kinetic models are available. Therefore, rather than arbitrarily plugging kinetic parameters in our simulation, we decided to back‐calculate the parameter values from KM and *V*_max_ values available from the literature [29]. This way, we can constrain and provide bounds on at least some kinetic parameters. For the reactions where we cannot determine the parameters, we use the values from the CRNT toolbox.

Briefly, we explain the way we back‐calculated to get the kinetic parameters. *V*_max_ and KM values are available for some of the reactions of our model from the database (https://www.ncbs.res.in/faculty/bhalla‐constant‐and‐database). For example, many reactions in this work follow the sequence of standard enzymatic reactions like the one given below.

$$E+S\overset{k_{1}}{\underset{k_{-1}}{⇌}}C\overset{k_{2}}{\to }E+P,$$

with *V*_max_ = *k*_2_ × ET, where ET is the total enzyme concentration. Since *V*_max_ values are available from the database, and to get *k*_2_, we fixed the ET value in our model. Once we know *V*_max_ and ET, we get *k*_2_ = *V*_max_/ET. To get other constants, *k*_1_ and *k*_−1_, we use the KM value. KM is (*k*_2_ + *k*_−1_)/*k*_1_, and since we do not know *k*_1_ in our case, we use the value obtained from the CRNT toolbox. This fixed the values of *k*_1_, *k*_2_, and KM. We finally get *k*_−1_ from (KM × *k*_1_) − *k*_2_.

For the unavailable parameters, we use the values we obtained from the CRNT toolbox. In this way, out of 48 parameters we could estimate 23 parameters by back‐calculation, which are from the enzymatic reactions. We provide all the details in Appendix S4. For all the simulations (except Fig. 7, where we used only CRNT values), we used the mix of kinetic constants obtained from estimates and the CRNT toolbox given in Table 2. In Table 1, we provide the kinetic parameters obtained from the CRNT toolbox.

## Results

### Bifurcation analysis with Cyr1 total as the bifurcation parameter: Identification of checkpoint transitions

**Fig. 2:**
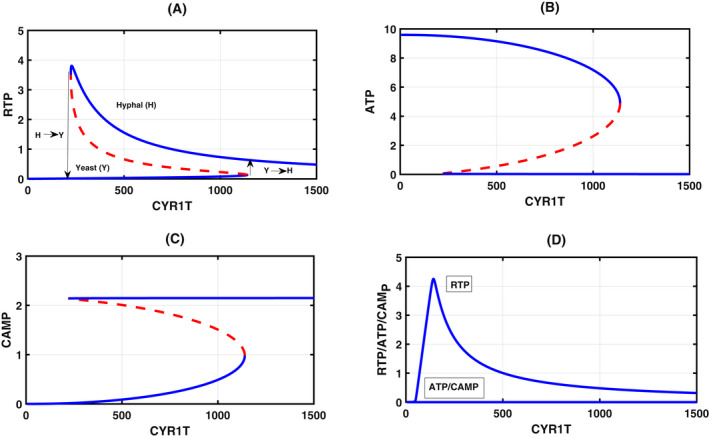
Bifurcation diagram: One parameter bifurcation diagram with Cyr1T as the bifurcation parameter and RTP (A), ATP (B), and CAMP (C) as steady‐state dynamical variables. Blue and broken red lines indicate the stable node and unstable saddle steady‐states. The meeting point of these lines is a saddle‐node bifurcation, where the switch from the yeast‐to‐hyphae (Y‐H) transition or another way (H‐Y) takes place. (D) Bistability is lost in the absence of ATP production (kf12 = 0), although it is biphasic. ATP and CAMP values are zero. Table 1 provides the value of kinetic constants. The other total concentration fixed in constructing the bifurcation parameters are Ira2T = 50, CD25T = 4, RasT = 100, PDE1T = 5, PDE2T = 5, CT = 50, RT = 50, PPAT = 5. We have provided the xppaut file in Appendix S5.

We start by fixing the total value of various species in the conservation relationships. Since our interest lies in determining the role of total Cyr1 (Cyr1T) and Ira2 (Ira2T) in morphological switching, we first fixed Ira2T and took Cyr1T as the bifurcation parameter to assess the steady‐state levels of RTP, ATP, and CAMP (Appendix S5, estimated + CRNT parameters; Appendix S6, only CRNT parameters). The bifurcation diagram shown in Fig. 2A–C exhibits the toggle switch. In Fig. 2A, an increase of Cyr1T results in a slow rise of RTP, as most of the Cyr1 is bound to Ira2 to form a complex (reaction R5). A discontinuous jump to high RTP levels happens once the complex is titrated (reaction R10). We attribute this discontinuous jump to the checkpoint in our model for the yeast‐to‐hyphal (Y‐H) transition and its reverse to the hyphal‐to‐yeast transition (H‐Y). After the transition, the RTP level gradually decreases because most of the free inactive Cyr1 gets activated by forming a complex with RTP (reaction R7). The complex then converts ATP to cAMP (reaction R8). This corresponds to a decrease in the ATP level, as shown in Fig. 2B, and the increase in cAMP level (Fig. 2C). Importantly, as indicated by the CRNT toolbox, in the absence of ATP production (taken kf12 = 0), bistability is lost (Fig. 2D), and as a result, the concentration levels of ATP and cAMP reduce to zero. However, the RTP is biphasic due to the initial conversion of RD to RTP by cdc25. Then the drop that follows is due to the formation of the C3 complex with Ira2 to form RD. cAMP is absent due to the absence of ATP in the system.

To explain the reverse transition, Lindsay et al. [30] showed the occurrence of reverse hyphal to yeast transition. When they treated *C. albicans* with farnesol, hyphal formation attenuated due to Cyr1 inhibition. Their experiments also indicated the involvement of the Ras signaling pathway in the H → Y transition with lower Cyr1 concentration (Fig. 2A). ATP abundance is unknown, but we believe that intracellular ATP must have accumulated, since the conversion of ATP → cAMP by Cyr1 may have become attenuated. Therefore, we speculate that ATP abundance must have been high, total Cyr1 must be low, and the hyphal to yeast transition must have been facilitated in the presence of farnesol.

### Antagonistic relationship between Ira2 and Cyr1

**Fig. 3:**
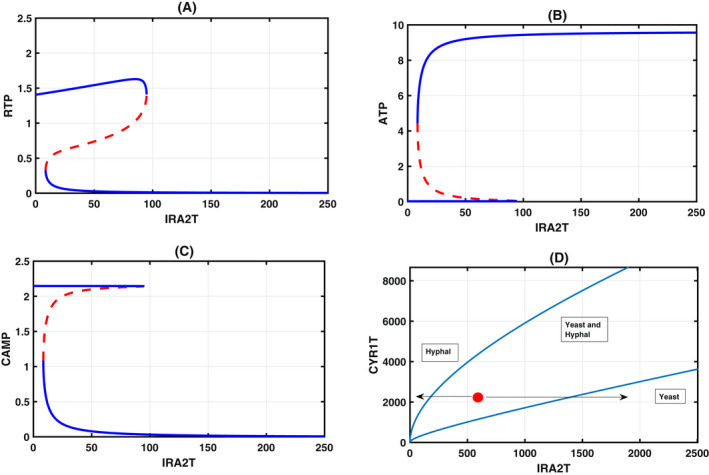
Bifurcation diagram: One parameter bifurcation diagram with Ira2T as the bifurcation parameter and RTP (A), ATP (B), and CAMP (C) as steady‐state dynamical variables. (D) Two‐parameter bifurcation diagram with total Ira2 as the second parameter. Two cusp bifurcations are seen. The red dot is inside the boundary of the cusp where both normal yeast and hyphal morphology coexist. Outside the left boundary, predominantly the hyphae form exists, while on the right boundary with low Cyr1 total, the yeast form exists. Table 1 provides the values of the kinetic constant. The other total concentration fixed in constructing the bifurcation parameters are Cyr1T = 500, CD25T = 4, RasT = 100, PDE1T = 5, PDE2T = 5, CT = 50, RT = 50, PPAT = 5.

According to our model, both Ira2 and ATP compete for Cyr1. If we use the total Ira2 as a bifurcation parameter, then we expect to observe a reverse trend when we compare it to the Cyr1 bifurcation diagram shown in Fig. 2. As expected, we see the reverse trend in our model (Fig. 3A–C) for a fixed total Cyr1. A two‐parameter bifurcation diagram shown in Fig. 3D in the Ira2‐Cyr1 parameter plane exhibits cusp bifurcation. We mark the regions of yeast, hyphal form, and the existence of both forms in the parameter plane. Inside the cusp, both forms coexist. Depending on the environmental conditions, they disengage one another and cross the cusp boundary through either one of the sides. The cusp boundaries on both sides are the checkpoint for the yeast‐to‐hyphal or the reverse of it in the CYR1T‐IRA2T parameter plane. Thus, our model captures the antagonistic role of Ira2 and Cyr1 in creating a bistable switch with ATP facilitating this process. In the next section, we further explore the effect of ATP production in this regulation.

### Effect of ATP production constant kf12

**Fig. 4:**
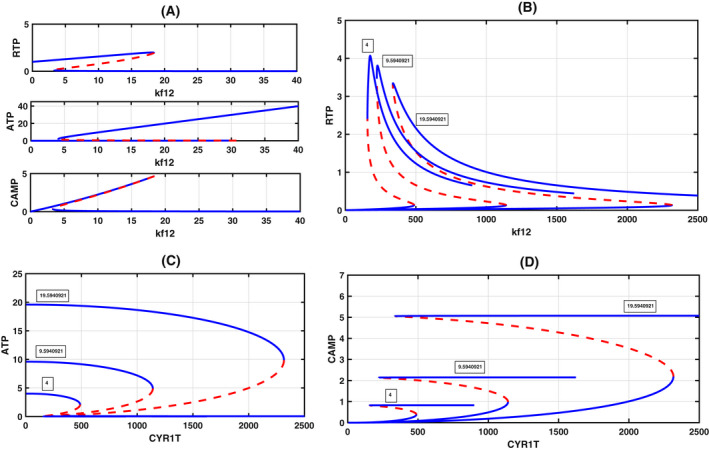
Bifurcation diagrams on the effect of ATP rate constant kf12 on the dynamics. (A) One parameter bifurcation diagram indicates a rapid change in ATP and cAMP with kf12 as a bifurcation diagram keeping Cyr1T = 500 and Ira2T = 50. (B–D) Delay in the saddle‐node bifurcation with the increase of kf12 for RTP, ATP, and CAMP with Cyr1T as the bifurcation parameter. Three kf12 values are chosen and they are kf12 = 4 (left), kf12 = 9.5940921 (middle, taken as standard), and kf12 = 9.5940921.

In experiments, since ATP plays a decisive role in triggering a switch in the Ras pathway [8], we constructed a one‐parameter bifurcation diagram with ATP production constant kf12 as the bifurcation parameter by keeping the total Ira2 and Cyr1T constant (Fig. 4A). An increase in the ATP production constant increases the RTP levels and leads to a rapid increase in the levels of both cAMP and ATP. We reason out the occurrence of a switch through the reaction steps provided in the earlier section. We start with reaction R10, where the increase in ATP production results in a quick complex formation, C5 (Ira2 + Cyr1; R10). This complex rapidly produces C6 (ATP + Cyr1) and Ira2. First, complex C6 quickly binds to RTP to form complex C7 to convert ATP to CAMP. Second, Ira2, another product, counteracts this downstream regulation. Now the fate of Ira2 is twofold; First, it forms a complex with RTP (reaction R3) and converts it to RD. This reaction reduces RTP availability. Second, it captures Cyr1 to form a complex C5 (reaction R5). As a result, Cyr1, in the absence of RTP availability, is inactive and cannot convert ATP to cAMP (reaction R7), and further, Cyr1 is not freely available due to reaction R7 to undergo any further reaction. Therefore, a tug‐of‐war goes on between Ira2 and Cyr1 to capture RTP. However, in the presence of excess ATP, Cyr1 wins over Ira2 in creating the switch. These two reactions are the leading cause contributing to the toggle switch with the increase of the ATP reaction constant kf12. We also expect this tug‐of‐war to delay the bifurcation (increase in threshold) with ATP increase. Thus, we also construct a bifurcation diagram for constant kf12 values, with Cyr1T as a bifurcation parameter. We expect the saddle‐node bifurcation to delay. As expected, our model shows a delay in saddle‐node bifurcation (Fig. 4B–D), and thereby the increase of threshold happens. Our model, therefore, predicts that increasing kf12 delays the switching to the hyphal form.

**Fig. 5:**
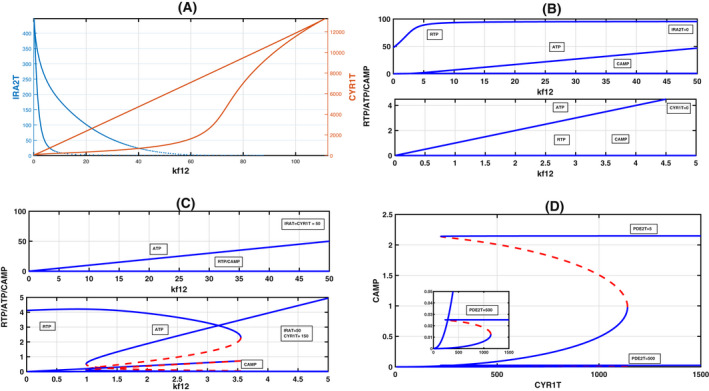
(A) Two‐parameter bifurcation diagram in kf12‐Ira2T and kf12‐Cyr1T parameter plane that exhibits cusp bifurcation. To simulate, we took Ira2T = 15 and Cyr1T = 500, keeping the other parameters constant. (B) In the top subplot, Ira2T = 0 and Cyr1T = 5. Bistability is absent. RTP and ATP are at high levels. CAMP is zero. In the bottom subplot, CYR1T = 0, IRA2T = 50. Here also bistability is absent. However, when both RTP and cAMP are zero, ATP remains high. (C) In the top subplot, both Ira2T = Cyr1T = 50. Again, bistability is absent. In the bottom subplot, Cyr1T = 150 = 3 × Ira2T (= 50). Bistability is present. The inset plot shows the magnification of the ATP and cAMP bifurcation diagram. The condition Cyr1T ≫ Ira2T is important for the presence of bistability by keeping the other parameters constant. See main text for explanation. (D) Increase in PDE2T, a high‐affinity phosphodiesterase‐2 from 5 to 500, results in a lowering of the cAMP level. There is no change seen in the bifurcation points. However, RTP and ATP steady‐state values remained the same (not shown) since PDE2 is a downstream effector of only cAMP.

The above argument also indicates that it is necessary to maintain a certain ratio of Ira2 and Cyr1 for the model to exhibit bistability. To assess this, we construct a two‐parameter bifurcation diagram with kf12 as the main parameter to determine the effect of Ira2 and Cyr1T on the bistable regime. In Fig. 5A, we project the two‐parameter bifurcation diagram of both the Ira2T‐kf12 and Cyr1T‐kf12 parameter plane. Both exhibit cusp bifurcation and have a wide cusp regime. Bistable systems with positive feedback loops are not robust, unlike negative feedback loops, in the sense that a small change in the bifurcation parameter may destroy three steady‐states to 2 to 1 [31]. In the present case, as the two‐parameter bifurcation diagram indicates, even a large variation in Ira2T or Cyr1T interactions with ATP will not drastically alter the dynamics, and variations in the parameters will still preserve bistability. We also found three interesting cases regarding the changes in Ira2T and Cyr1T parameter values: (a) If Ira2T is absent (Ira2T = 0), as one expects, RTP, ATP, and CAMP are present at high levels. However, bistability is lost. On the other hand, again, as one expects, if Cyr1T is absent (Cyr1T = 0), then RTP and CAMP levels become zero and ATP, although it is present in excess, cannot carry out the transition switch because of the Cyr1T absence (Fig. 5B). (b) If Ira2T and Cyr1T values are the same (Ira2T = Cyr1T = 50) or very close to each other, again bistability is lost (Fig. 5C, top subplot). We found that Ira2 interacts strongly with RTP and converts to RD in this case. ATP is present in excess and interacts with Cyr1T to form a complex C6. Since RTP is unavailable, complex C6 cannot bind to RTP; therefore, cAMP production is zero. Therefore, this points to a weak or no interaction between Ira2 and Cyr1 and bistability is lost. (c) If Cyr1T is present more than Ira2T, bistability is always present. Thus, for the model to exhibit bistability, with kf12 as the bifurcation parameter, Cyr1T ≫ Ira2T (Fig. 5D, bottom subplot).

### Effect of phosphodiesterases 1 and 2 on cAMP levels

We simulated the role of phosphodiesterases in regulating cAMP levels in the model. Phosphodiesterases, the downstream regulators, involve in a negative feedback loop that controls the cAMP production by converting it to AMP. At higher levels of PDE‐1 and PDE‐2, they strongly reduce the cAMP levels without affecting RTP and ATP levels (Fig. 5D). PDE‐1 and PDE‐ 2 can be thought of as other controllers to reduce virulence. For example, the negative feedback regulation of CAMP by PDE2 is important to induce the yeast to hyphal transition [30]. Farnesol is known to induce the H → Y transition, but a mutated PDE2 cannot regulate cAMP to suppress this transition, and as a result, Y → H transition takes place. Therefore, PDE2 negative feedback in *C. albicans* is important to arrest the Y → H transition and facilitate the H → Y transition. We know that PKA regulates cAMP directly and through Ira2 and Cdc25, present upstream of the Ras pathway. This regulation creates feedback loops and generates complex dynamics [23]. However, presently we are interested in switch‐like dynamics and do not consider other feedback regulations that give rise to oscillations.

## Discussion

Before we proceed further, we would like to explain why we hypothesize a bistable dynamic for yeast‐to‐hyphae (*Y* → H) and the reverse hyphae‐to‐yeast (*H* → Y) transitions. The (*Y* → H) and (*H* → Y) transitions happen in a switch‐like fashion. The switch involves a threshold that controls the transition, and the threshold is taken as a checkpoint that determines whether the transition will occur or not.

We used the word checkpoint from the Grahl et al. article [8]. They explained the role of ATP as an intracellular pool that serves as a “checkpoint” in Ras1 signaling under hypha‐inducing conditions. Their study also highlighted the regulation of hyphal transition by IRA2, CYR1, and ATP in the Ras pathway. In fig. 9 of their article, they provided a general idea about the interplay of these proteins. Further, Silao et al. [3], in their work, commented on Grahl et al.'s. fig. 9 on Ras network that the AC interacts with other proteins to give rise to positive feedback, with ATP as input.

However, it was not apparent how the positive feedback network arises from the interaction among CYR1‐ATP‐IRA2. With the help of the Grahl et al. cartoon in fig. 9 of their article, and Silao et al. work the present work explains how positive feedback acts as a checkpoint in yeast‐to‐hyphal transition. We began this by building a network and proposing a new set of reactions based on the network structure. Again, it is difficult to tease out positive feedback and infer the dynamics from the constructed network. Therefore, we performed CRNT analysis on this network. The analysis indicated that our network could exhibit bistability. We also know that positive feedback in the network is one of the criteria for bistability for the choice of kinetic parameters. The positive feedback loop in our network structure is unusual. The feedback loop is not a traditional mutual inhibition or activation or autoregulatory, where tracing the edges provides a clear idea about the feedback loops. We may categorize it as noncanonical, along the lines of Li et al.'s miRNA–mRNA interactions model [18]. However, we have not conducted an extensive analysis in the present work and relegated the feedback loop analysis in the network structure as future work.

Putting all these pieces of information together, we concluded that the Ras pathway might exhibit bistability. We attributed the bistability to– *Y* → H transition and *H* → Y transitions.

**Fig. 6:**
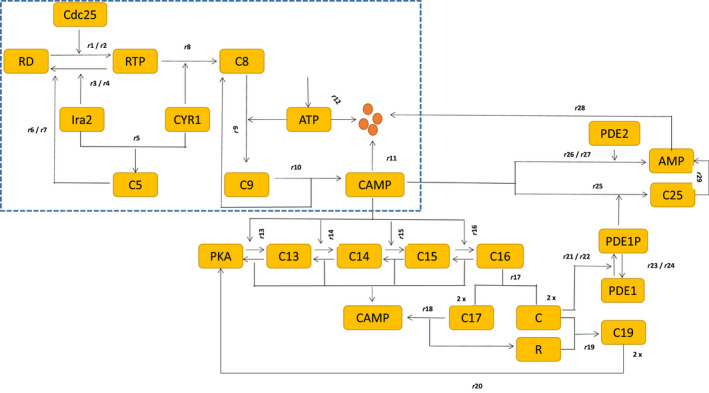
Biological circuit of Ras‐GTP pathway for model‐2. Yellow boxes denote the proteins with their names. We first start from the top left of the circuit inside the dotted box with RD, a short form for Ras‐GDP and RT for Ras‐GTP. The circuits inside the dotted box show the newly proposed reaction for the interaction between Ira2 and Cyr1, ATP, and Cyr1, which plays an essential role in forming cAMP. Subsequently, the other module outside the box is the derepression of PKA by cAMP that releases the catalytic C part of the PKA and downregulates cAMP through phosphodiester Rases PDE‐1 and 2 to form a negative feedback loop. The *ri*'s are the corresponding rates of the reaction based on mass action kinetic laws. In this network, Ras‐GDP is converted to RT, the Ras‐GTP, by the enzyme cdc25. The enzyme Ira2 carries out the reverse reaction. Cyr1, the enzyme adenyl‐cyclase, binds to Ira2 to form a complex C5 to arrest the conversion of Ras‐GTP to Ras‐GDP, and thereby reduce its activity. Cyr1 also binds to RTP, which forms an inactive complex C8. This inactive complex binds to ATP to form a trimer C9 to activate Cyr1 and facilitates cAMP formation. The rest of the details about PKA interaction with CAMP are the same as provided in the caption of Fig. 1.

Although we have provided the Ira2‐Cyr1‐ATP interactions as the backbone of the main module in the network that created the bistable switch and confirmed by the CRNT toolbox, the question is whether this network is unique or are there any other plausible network connections that can explain the occurrence of the switch? To answer this, we built another network by tinkering with the network model in Fig. 1. We show that the tinkered network in Fig. 6 and below, we provide the corresponding biochemical steps. We have provided the .NET (Appendix S7) and .ode files (Appendix S8) in the Supporting Information. We call this model‐2 and provide only the results from the bifurcation simulation.

The biochemical steps for the model‐2 are as follows:

$R1:\;\mathrm{RD}+\mathrm{CD}25\overset{\mathrm{kf}1}{\underset{\mathrm{kr}1}{⇌}}C1\;r1=\mathrm{kf}1*\mathrm{CD}25*\mathrm{RD}-\mathrm{kr}1*C1,$

$R2:\;C1\overset{\mathrm{kf}2}{\to }\mathrm{RTP}+\mathrm{CD}25\;r2=\mathrm{kf}2*C1,$

$R3:\;\mathrm{RTP}+\mathrm{Ira}2\overset{\mathrm{kf}3}{\underset{\mathrm{kr}3}{⇌}}C3\;r3=\mathrm{kf}3*\mathrm{RTP}*\mathrm{Ira}2-\mathrm{kr}3*C3,$

$R4:\;C3\overset{\mathrm{kf}4}{\to }\mathrm{RD}+\mathrm{Ira}2\;r4=\mathrm{kf}4*C3,$

$R5:\;\mathrm{Ira}2+\mathrm{Cyr}1\overset{\mathrm{kf}5}{\underset{\mathrm{kr}5}{⇌}}C5\;r5=\mathrm{kf}5*\mathrm{Ira}2*\mathrm{Cyr}1-\mathrm{kr}5*C5,$

$R6:\;C5+\mathrm{RTP}\overset{\mathrm{kf}6}{\underset{\mathrm{kr}6}{⇌}}C6\;r6=\mathrm{kf}6*C5*\mathrm{RTP}-\mathrm{kr}6*C6,$

$R7:\;C6\overset{\mathrm{kf}7}{\to }\mathrm{RD}+C5\;r7=\mathrm{kf}7*C6,$

$R8:\;\mathrm{RTP}+\mathrm{Cyr}1\overset{\mathrm{kf}8}{\underset{\mathrm{kr}8}{⇌}}C8\;r8=\mathrm{kf}8*\mathrm{RTP}*\mathrm{Cyr}1-\mathrm{kr}8*C8,$

$R9:\;C8+\mathrm{ATP}\overset{\mathrm{kf}9}{\underset{\mathrm{kr}9}{⇌}}C9\;r9=\mathrm{kf}9*C8*\mathrm{ATP}-\mathrm{kr}9*C9,$

$R10:\;C9\overset{\mathrm{kf}10}{\to }\text{CAMP}+C8\;r10=\mathrm{kf}10*C9,$

$R11:\;\text{CAMP}\overset{\mathrm{kf}11}{\to }0\;r11=\mathrm{kf}11*\text{CAMP},$

$R12:\;0\overset{\mathrm{kf}12}{\underset{\mathrm{kr}12}{⇌}}\mathrm{ATP}\;r12=\mathrm{kf}12-\mathrm{kr}12*\mathrm{ATP}.$

The rest of the reactions (R13–R29) are the same as those given earlier in model‐1 for downstream PKA derepression and negative feedback regulation.

We first briefly explain the main difference between the two‐network construction. In Fig. 6, unlike in Fig. 1, Cyr1 binds to Ira2 to form a complex C5 to facilitate the conversion of Ras‐GTP to Ras‐GDP and thereby reduce the RTP activity. We base our network construction on the fig. 9 influence diagram of [8], where they show that Ira2 does not interact directly with RTP but through the complex C5 under the ATP depleted condition. Further, we also assume that the Cyr1 acts like a scaffold by first recruiting RTP directly to form an inactive complex C8, and it follows it up by accommodating ATP as and when it is available. Once this assembly is complete, Cyr1 becomes active enough to convert ATP to cAMP. Here, the sequence of binding is different from the earlier reaction steps. Except for this difference in reaction steps, this network in principle follows the exact antagonistic reaction between Ira2 and Cyr1, as shown in Fig. 1.

**Fig. 7:**
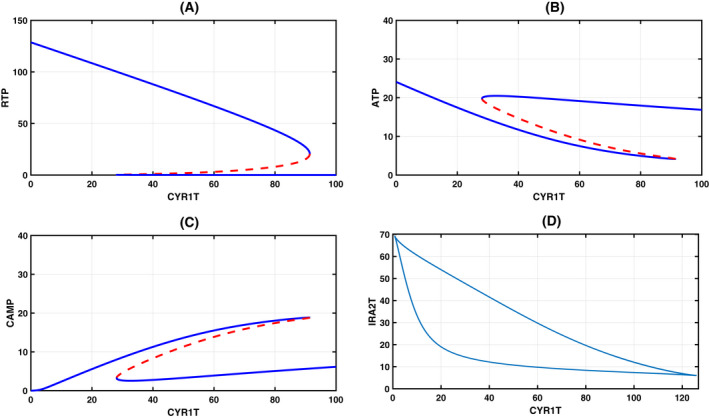
Bifurcation diagram for model‐2: One‐parameter bifurcation diagram with Cyr1T as the bifurcation parameter and RTP (A), ATP (B), and CAMP (C) as steady‐state dynamical variables. (D) Two‐parameter bifurcation diagram with total Ira2 as the second parameter. Two cusp bifurcation is seen. The total conserved species are Ira2T = 15, CD25T = 10, RasT = 150, PDE1T = 5, PDE2T = 5, CT = 5, RT = 5, PPAT = 5. The xppaut file is given in Appendix S8.

To see model‐2's behavior, we again construct one‐ and two‐parameter bifurcation diagrams with Cyr1T as the bifurcation parameter. We use the kinetic constants from the CRNT toolbox to build the bifurcation diagrams. The bifurcation diagram is given in Fig. 7 and the trend is reversed with respect to Fig. 2. Here, cAMP transitions from a high‐to‐low level when Cyr1T increases, but we expect the opposite trend for the yeast‐to‐hyphal transition. Therefore, we discard this network without proceeding further with the numerical simulations.

Finally, there are three transitions in normal to virulence transitions; yeast to pseudo‐hyphae to hyphae. Presently the signaling pathway and regulatory mechanisms are unknown about how tristability can happen. We know the existence of tristable dynamics from a gene regulatory network point of view in the white‐opaque‐gray transition [32], but have yet to elucidate their signaling mechanism. We will explore this aspect once we obtain sufficient clarity about the Ras pathway on pseudo‐hyphae.

## Conclusion

In this work, we have captured the specific important reaction steps of the Ras pathway to determine the cause of yeast‐to‐hyphal transitions in *C. albicans* in the presence of the energy sensor, ATP. Writing a stepwise elementary reaction for the protein interactions helped us to identify the cause of switching in *C. albicans*. The approach of going from the biological circuit → molecular mechanism → ODE model → bifurcations → biological meaning is rife with many difficulties [10]. First, the number of nodes in the network becomes quickly unmanageable, and the inclusion of feedback interactions creates further complexity. Sabouri‐Ghomi et al. [33] suggest that the modular approach is the best option to circumvent these problems by which one starts with a minimal model with a finite number of nodes and well‐known directed interactions that capture not only the essential dynamics of the system but also help to estimate the realistic kinetic parameters of the model. In the present case, we followed this approach and built a minimal model of *C. albicans*. There are two modules in our model. The first module is the Ras regulation by Cyr1 and Ira2 in the presence of ATP to produce the final product, cAMP. The second module involves the downstream regulation of cAMP by PKA through negative feedback loops. Despite taking the modular route, and only the first module that creates a bistable switch, our circuit is complex with 29 biochemical steps, 10 corresponding ODEs, and conservation relationships. Although modeling by MM kinetics may have reduced the number of variables and parameters and possibly would have obtained the same result, this may be inappropriate [34]. Moreover, if we have resorted to MM type of modeling and obtained switch‐like dynamics, the mechanistic details about the occurrence of bistability may become hidden, and to tease out the details, unpacking has to be done (see Ref. [33] for examples and discussion). Unpacking the MM type of ODEs also requires considerable understanding and expertise about the system, and this will only further complicate the modeling process (see Ref. [33] for multiple ways to generate trimers when they unpack the mechanism of the cell cycle model). Therefore, although elaborate and sometimes cumbersome, we resolved to start our modeling process with the mass action type kinetic models. Another problem arising in our modeling is the nonavailability of kinetic constants. A complex circuit with many kinetic constants may exhibit many interesting dynamics for a combination of parameter sets, but it may be irrelevant to the problem at hand. For example, although the Ras pathway can exhibit oscillatory dynamics [23], it is not necessary here, since we are interested in understanding the switching phenomena. Thus, we focused only on the bistable dynamics. We used CRNT to relate the network structure to dynamics with this aim in mind. Again, we could develop many network structures, two of which we have discussed, indicating the nonuniqueness of the reaction mechanism, and this problem can be resolved only through experiments. Nevertheless, we hope that our set of biochemical reactions and dynamical model may provide insight into the occurrence of switching behavior in *C. albicans*.

## Conflict of interest

The authors declare no conflict of interest.

## Author contributions

SK conceived the study, performed the experiments, analyzed the data, and wrote the article.

## Supporting information


**Appendix S1.** Matlab file get a conservation relationship from the stoichiometric matrix (Appendix S2), The output from the Matlab file gives the linearly dependent variables. This is cut and pasted in the Appendix S2 file in the second sheet. This gives the conservation relationship.

**Appendix S2.** The stoichiometry matrix for MAIN_MODEL‐1.

**Appendix S3.** The.NET CRNT file for Model‐1.

**Appendix S4.** The reaction parameter estimates are given in the file.

**Appendix S5.** Xppaut file used for all the simulations. This contains the estimated parameters as well as CRNT parameters to simulate Figs. 2‐5.

**Appendix S6.** XPPAUT file of the primary model to get all the bifurcation diagrams with only CRNT parameters.

**Appendix S7.** The.NET CRNT file for Model‐2.

**Appendix S8.** XPPAUT file to get the bifurcation diagram given in Fig‐7.

## Data Availability

All data are included in the article.
