# Supplementary material for: A mathematical model captures the role of adenyl cyclase Cyr1 and guanidine exchange factor Ira2 in creating a growth‐to‐hyphal bistable switch in Candida albicans
Source: FEBS Open Bio. 2022 Aug 30;12(10):1700–16. doi: 10.1002/2211-5463.13470 (PMC9527597; doi:10.1002/2211-5463.13470)
Supplement: Supplementary file 4 — Appendix S4. The reaction parameter estimates are given in the file. [file FEB4-12-1700-s005.docx]

Supplemetary: Parameters from the database

<https://www.ncbs.res.in/faculty/bhalla-constant-and-database>

Bhalla, U., Iyengar,R., 1999. Emergent Properties of Networks of Biological Signaling Pathways. Science, 283, 381-387.

1. Consider the reactions R1-R4 in the main text.

# R1: RD + CD25 <---------> C1 r1=kf1*CD25*RD-kr1*C1

# R2: C1 ------> RTP + CD25 r2 = kf2*C1

# R3: RTP + IRA2 <---> C3 r3= kf3*RTP*IRA2- kr3*C3

# R4: C3 ---->RD + IRA2 r4= kf4*C3

To get kinetic constants, we first fixed all the total quantities present in the mass conservation relationships. These are CYR1T=5, IRA2T=50, CD25T=4, RAST=100

PDE1T=5, PDE2T=5, CT=50, RT=50, and PPAT=5

The reactions R1 and R2 are in the MM form. Parameter KM and VMAX of these reactions are given in terms of kinetic constants below.

KM12 = (kr1 + kf2)/kf1; VMAX12 = kf2*CD25T

Similarly, for reactions R3 and R4, we have

KM34 = (kr3 + kf4)/kf3; VMAX34 = kf4*IRA2T

KM12, KM34, VMAX12, and VMAX34 are got from [ Bhalla and Iyengar, (1999), <https://www.ncbs.res.in/faculty/bhalla-constant-and-database].>. The reaction scheme-B, and the reaction-10, from the database gives the conversion of RAS-GDP to RAS-GTP by GEF as follows. The parameters KM (we call KM12) and VMAX (we call it VMAX12) are also given. This is given below as

GDP-RAS ----GEF---> GTP-RAS KM12=0.50505; VMAX12=0.02

Here GEF is CDC25. Our reaction is based on mass action kinetics. So we back calculate the kinetic constants by fixing total CD25T = 4. Then the kf2 value is

VMAX12 = kf2*CD25T, and so kf2 = VMAX12/CD25T = 0.02/4 = 0.0050.

We also know KM12 = 0.50505 = (kr1 + 0.0050)/kf1. Rather than guessing, we choose kf1 = 2.6702E-2 got from CRNT toolbox. This gives

0.50505 = (kr1 + 0.0050)/2.6702E-2

0.50505*2.6702E-2 = kr1 + 0.0050;

0.0135 = kr1 + 0.0050

kr1 = 0.0085.

The kinetic constants for reactions R1-R2 are

R1: kf1 = 2.6702E-2, kr1 = 0.0085,

R2: kf2=0.0050

1. Similarly for R3 and R4,

# R3: RTP + IRA2 <---> C3 r3= kf3*RTP*IRA2- kr3*C3

# R4: C3 ---->RD + IRA2 r4= kf4*C3

We back calculate all the kinetic constant of reactions R3-R5, from the constant provided in the website [Science, Bhalla, <https://www.ncbs.res.in/faculty/bhalla-constant-and-database].> for the following reaction .

GTP-RAS ----GAP---> GDP-RAS KM34=1.0104; VMAX34=10

From the above kinetic constants, we back-calculate to get kinetic constants for our model. For that we fixed IRA2T = 50, and from CRNT toolbox, kf3=0.20554648. Then,

KM34 = (kr3 + kf4)/kf3; VMAX34 = kf4*IRA2T

kf4 = VMAX34/IRA2T = 10/50 = 0.2000

KM34 = 1.0104 = (kr3 + 0.2000)/0.20554648

1.0104*0.20554648 = kr3 + 0.2000

0.2077 = kr3 + 0.2000

kr3 = 0.0077.

The kinetic constant for reactions R3-R4 are

# R3: kf3=0.2055468, kr3=0.0077

# R4: kf4 = 0.2

3. For the following reactions of cAMP with PKA,

# R13: CAMP + PKA<--->C13 r13=kf13*CAMP*PKA-kr13*C13

# R14: C13 + CAMP <---> C14 r14=kf14*C13*CAMP-kr14*C14

# R15: C14 + CAMP <---> C15 r15=kf15*C14*CAMP-kr15*C15

# R16: C15 + CAMP <---> C16 r16=kf16*C15*CAMP-kr16*C16

# R17: C16 <----> 2C + 2C17 r17=kf17*C16-kr17*(C^2)*(C17^2)

# R18: C17 ------> R + CAMP r18=kf18*C17

# R19: R + C <----> C19 r19=kf19*R*C-kr19*C19

# R20: 2*C19 <----> PKA r20=kf20*(C19^2)-kr20*PKA

We consider the following kinetic constants from reaction scheme J from database <https://www.ncbs.res.in/faculty/bhalla-constant-and-database].>

R13: kf13=0.00009;kr13=33;

R14: kf14=0.00009;kr14=33;

R15: kf15=0.000125; kr15=110;

R16: kf16=0.000125; kr16=32.5;

R17: kf17=60; kr17=0.00003;

R18: Not known

R19: Not known

R20: kf20=60;kr20=0.00003;

1. The cAMP regulation by PDE-1 and PDE-2 is

# R21: C + PDE1 <---> C21 r21=kf21*C*PDE1 - kr21*C21

# R22: C21----> PDE1P + C r22=kf22*C21

We fixed total PJA as PKAT = CT + RT = 50 + 50 =100; So, CT = 50; and for kf21=5.0883E-2, we took it from CRNT toolbox.

From reaction scheme-C, reactions 12 and 13, [Science, Bhalla, <https://www.ncbs.res.in/faculty/bhalla-constant-and-database].>

we get KM12=7.5, VMAX12=9;

As above, we use

VMAX12= 9; so VMAX12 = kf22*CT and so kf22 = VMAX12/CT = 9/50 = 0.18

KM2123 = 7.5 = (kr21+0.18)/kf21. = 7.5 = (kr21 + 0.9)/5.0883E-2

This gives the following kinetic constants for reactions R21-R22.

R21: kf21 = 5.0883E-2; kr21 = 0.2016;

R22: kf22 = 0.18

1. For the reactions R25 and R29,

# R25: PDE1P + CAMP <---> C25 r25=kf25*CAMP*PDE1P - kr25*C25

# R29: C25 ----> AMP + PDE1P r29=kf29*C25

We assume that little PDE1P is formed from PDE1T and therefore, we take PDE1P ~ PDE1T = 5; We also take kf25=1.92285863 from CRNT toolbox.

From reaction scheme-C, reaction-15, [Science, Bhalla, <https://www.ncbs.res.in/faculty/bhalla-constant-and-database].> we have KM15 = 19.841, VMAX15=20

kf29 = VMAX15/PDE1T = 20/5 = 4;

KM15 = (kr25 + kf29)/kf25

19.841 = (kr25 + 4)/1.9228563;

kr25 = 34.1514

This gives the kinetic constants reactions R25 and R29.

R25: kf25 = 1.9228563, kr25=34.1514,

R29: kf29=4;

1. Finally, PDE-2 has two-fold high affinity for cAMP than PDE-1. So, for the reactions R26 and R27, we took 2 times both kf25 and kf27.

# R26: CAMP + PDE2 <----> C26 r26=kf26*CAMP*PDE2-kr26*C26

# R27: C26 ------> AMP + PDE2 r27=kf27*C26

R26: kf26=2 x kf25 = 4, kr26=34.1514 (no change in reversible constant )

R27: kf27=2 x kf29 = 8;

Therefore, kinetic constants we used in the simulation are

######From estimate ########

par kf1=2.6702E-2

par kr1=0.0085

par kf2=0.0050

par kf3=0.20554648

par kr3=0.0077

par kf4=0.2000

###### From CRNT, new reaction specific to this work #############

par kf5=0.18971829

par kr5=2.6163E-2

par kf6=3.7537582

par kr6=7.4770E-2

par kf7=3.6846E-2

par kr7=0.1039351

par kf8=0.79413092

par kf9=12.486667

par kr9=2.7294819

par kf10=1.4802E-2

par kr10=4.0322E-3

par kf11=1

par kf12=9.5940921

par kr12=1

############ Estimates #############

par kf13=0.00009

par kr13=33

par kf14=0.00009

par kr14=33

par kf15=0.000125

par kr15=110

par kf16=0.000125

par kr16=32.5

par kf17=60

par kr17=0.00003

###########From CRNT ##########

par kf18=0.75147295

par kf19=4.7825E-2

par kr19=2.6692966

par kf20=60

par kr20=0.00003

#############Estimates ###############

par kf21=5.0883E-2

par kr21=0.2016

par kf22=0.18

##########From CRNT#########

par kf23=0.12346798

par kr23=0.49182469

par kf24=0.49182469

############Estimates ###############

par kf25=1.9228563

par kr25=34.1514

par kf29=4

par kf26=4

par kr26=34.1514

par kf27=8

#############From CRNT ###########

par kf28=1
